# The Emerging Role of PARP Inhibitors in the Treatment of Epithelial Ovarian Cancer

**DOI:** 10.1155/2010/151750

**Published:** 2009-12-16

**Authors:** Lilian T. Gien, Helen J. Mackay

**Affiliations:** Princess Margaret Hospital, University of Toronto, Toronto, ON, Canada M5G 2M9

## Abstract

Poly(ADP-ribose) polymerase-1 (PARP-1) is an important novel target in cancer therapy. This enzyme is essential in the repair of single-stranded breaks in DNA via the base excision repair pathway. Drugs which inhibit PARP are emerging as a promising new class of anticancer agents particularly effective against tumors which have lost homologous recombination (HR) through loss of functional BRCA1 and BRCA2. PARP inhibitors potentially represent a major breakthrough for patients with hereditary BRCA-associated cancers. Furthermore their role in sporadic epithelial ovarian cancer is emerging with identification of additional subpopulations of women who may benefit a priority. This paper will summarize the mechanism of action of PARP inhibition and its role in the treatment of BRCA1- and 2-associated cancers. We will then expand on the broader relevance and future directions for PARP inhibition in the clinical setting.

## 1. Introduction

Epithelial ovarian cancer (EOC) is the fifth leading cause of death in women in North America [1]. Despite the efficacy of platinum-based chemotherapy, over 75% of women with stage III/IV EOC ultimately relapse and die from their disease. Median survival for women whose disease does not respond or in whom duration of response is short is less than 12 months [2]. Traditional cytotoxics topotecan and liposomal doxorubicin demonstrate only modest efficacy in women with platinum resistant EOC and are associated with significant toxicity [3]. New therapeutic approaches, and the ability to identify patients groups who will derive benefit from them, are urgently required.

Over recent years the investigation of DNA repair in cancer cells has been a very active area of translational research. All cells have a number of overlapping pathways to protect the genome from DNA damage which occurs as a result of normal cell cycling, environmental insults, or cytotoxic chemotherapy. It is well recognized that when mutations occur within these DNA repair pathways there is an increased risk of malignant transformation and chemotherapy resistance [4]. Much research has focused on protecting cells from DNA damage and/or restoring DNA repair function. However, emerging data suggest that the concept of “synthetic lethality,” that is, exploiting the vulnerability of cancer cells which have lost one mechanism of DNA repair by targeting a second pathway, may be a particularly attractive therapeutic approach. Poly(ADP-ribose) polymerase (PARP) is an enzyme which pIays an important role in the recognition and repair of single-strand DNA breaks via the base excision repair (BER) pathway [5]. Over the last few years it has become apparent that in cells which have lost BRCA1 or BRCA2, components of a second DNA repair pathway, homologous recombination (HR), are particularly sensitive to PARP inhibition. These data suggest that PARP inhibitors may be particularly useful for the treatment of women with hereditary BRCA1/2-associated EOC [6, 7]. Targeted therapy using PARP inhibitors has become an important novel strategy for treating those with hereditary ovarian cancer. Furthermore the identification of other subpopulations of women with EOC who may benefit from this approach is an active area of research. 

This paper will outline the mechanism of PARP inhibition and discuss this in relation to loss of BRCA function. We will summarize the preclinical and clinical evidence from the most recent studies and discuss future directions for PARP inhibition in EOC.

## 2. BRCA1 and BRCA2

BRCA1 or BRCA2 mutations occur in 0.1–0.8% of the general population and are inherited in an autosomal dominant manner [8]. They are well recognized to have a higher incidence in certain ethnic groups, such as women of Ashkenazi Jewish descent [9]. Women carrying a mutation in BRCA1 have a lifetime risk of developing ovarian cancer of between 40 and 50%, while those carrying a BRCA2 mutation have a slightly lower risk of 10–20% [10]. Over the past ten years, the focus of management for those identified as BRCA1/2 mutation carriers has been on cancer prevention and early cancer detection. However, despite prophylactic measures to reduce risk of EOC, many BRCA1/2 carriers will already have cancer at the time their mutation is diagnosed.

The BRCA1 gene is located on chromosome 17q21, while BRCA2 is located on chromosome 13q12 [11, 12]. BRCA1 and BRCA2 play major roles in the repair of DNA double-strand breaks (DSBs) by homologous recombination (HR). HR repairs DSBs that occur in late S and G2 phase of the cell cycle and also has a key role in repairing DSBs that result from unrepaired single-strand break (SSB) [13]. BRCA1 signals the presence of DSBs, while BRCA2 is directly involved in the mechanism of HR. In the absence of BRCA1 or BRCA2, alternative DNA repair pathways are used, which result in chromosomal instability and cell death. Normal cells of carriers are usually heterozygote with loss of the second allele occurring during tumorigenesis in the tumor cells of these women [14].

Currently, the treatment of patients with BRCA-associated EOC is identical to those with sporadic EOC. However, even prior to the emergence of the PARP inhibitors, data suggested that cancers associated with BRCA mutations responded differently to chemotherapy [15]. Tan et al. compared 22 BRCA-positive patients with EOC to 44 nonhereditary EOC controls in a matched case-control study. They found that BRCA-positive patients have higher response rates to first line platinum-based treatment (81.8% versus 43.2%, *P* = .004), subsequent lines of platinum-based treatments (second line, 91.7% versus 40.9%, *P* = .004), longer tumor-free intervals between relapses, and improved overall survival (8.4 versus 2.9 years, *P* < .002) [16]. This data implies that different strategies may be required in this group of women.

## 3. Poly(ADP-Ribose) Polymerase Inhibitors

There are currently 17 members of the PARP superfamily identified [17]. PARP-1 is the most studied enzyme, which is involved in the repair of SSBs of DNA by the base excision repair (BER) pathway [5]. Targeting the nuclear enzyme PARP-1 represents a new and novel approach to the treatment of EOC and appears to be particularly promising for those carrying mutations in the BRCA1 and 2 genes [14]. 

**Figure 1:**
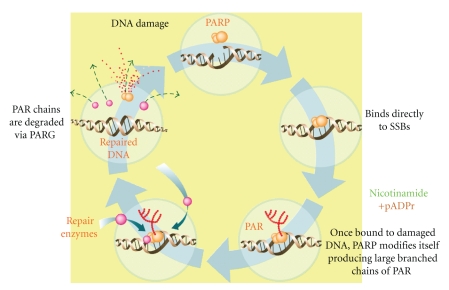
The role of PARP in the repair of single-strand DNA breaks via the base excision repair pathway.

Cells utilize several overlapping DNA repair mechanisms to maintain the integrity of the genome. PARP-1 activation occurs in response to metabolic, chemical, or radiation-induced DNA SSBs and forms part of the BER pathway [18, 19]. PARP-1 detects and signals the presence of an SSB by binding to DNA adjacent to the damage. Once bound, PARP-1 catalyzes the cleavage of the coenzyme nicotinamide adenine dinucleotide (NAD+) into nicotinamide and ADP-ribose to produce highly negatively charged branched chains of poly(ADP-ribose) (PAR). A multiprotein repair complex is then formed including repair enzymes DNA ligase III, the DNA polymerase pol B, and scaffolding proteins such as XRCC1 (X-ray repair cross-complementing 1). Following ADP-ribosylation, PARP-1 has reduced affinity for DNA and is released. After repair, the PAR polymers are degraded via poly(ADP-ribose) glycohydrolase (PARG) [14] (Figure 1). The role of PARP-1 may not be limited to just SSB repair; roles in the DSB repair response have also been proposed [20]. 

The new generation of PARP inhibitors inhibits PARP by competitive inhibition of NAD+. In the preclinical setting, PARP-1 inhibitors enhance the cytotoxic effects of ionizing radiation and cytotoxic chemotherapy [21]. Additionally, in the preclinical setting, the use of PARP-1 inhibitors as single agents did not cause any measurable toxicity, but the combination of PARP-1 inhibitor with temozolomide in the tumor-bearing mice caused significant toxicity [22]. There did not seem to be a correlation, however, between the antitumor activity and the toxicity of the PARP inhibitor-temozolomide combinations, suggesting that toxicity and chemosensitization were by different mechanisms. While promising in combination with other agents, PARP inhibitors appear to be particularly potent in patients who have defects in DNA repair. 

In a normal cell, PARP-1 inhibition leads to failure of SSB repair, resulting in the formation of a DSB in the DNA when a replication fork encounters the SSB. Thus the DSB can be repaired by HR and the fidelity of the genome maintained. However, in cells carrying defects in BRCA1/2, HR is defective, resulting in an attempted repair of the DSB by the more error prone nonhomologous end joining (NHEJ) pathway [18]. As a result, the cell acquires lethal levels of damage and cellular viability is lost, a prime example of “synthetic lethality” with the malignant cell able to function with the loss of one DNA repair mechanism (HR) but ceasing to be viable with the loss of a second (BER) [23, 24]. As most BRCA1/2 carriers have one normal allele, the hope was that inhibition of PARP would be selective for tumor cells.

In 2005, two preclinical papers demonstrated the sensitivity of BRCA1- and BRCA2-deficient cell lines to PARP inhibition [6, 7]. The first paper by Bryant et al. demonstrated reduced survival of BRCA2-deficient cell lines with four PARP inhibitors. They concluded that BRCA2-deficient cells were sensitive to PARP inhibition, and that monotherapy with one of these agents could selectively kill cancer cells [6]. In the same year, Farmer et al. demonstrated how both BRCA1- and BRCA2-deficient cells lines were sensitive to inhibition of PARP-1, and that BRCA2 deficient cells were more than 1000 times more sensitive to nanomolar concentrations of PARP inhibitor [7]. Both of these papers demonstrated how homozygotes (tumor cells) are sensitive to the mechanism of PARP inhibition; whereas heterozygotes (the rest of the patient's cells) are insensitive to this mechanism and should not exhibit toxicity. These findings from two independent groups using different chemical classes of PARP inhibitors on different BRCA-deficient cell lines were the first to suggest the potent effect of PARP inhibition.

## 4. Clinical Evidence in Phase I and II Trials

A number of PARP inhibitors have entered the clinic in both intravenous and oral formulations. The four which are furthest along in terms of development are AGO14699 (Pfizer), AZD2281 (AstraZeneca), ABT-888 (Abbott), and BSI-201 (BI Par), and all four of these compounds demonstrate profound inhibition of PARP-1. 

Olaparib (AZD2281, KU- 0059436, AstraZeneca) is an oral small-molecule PARP inhibitor. The first clinical evidence demonstrating the sensitivity of BRCA-mutated cancers to PARP inhibitor monotherapy was presented in a study by Yap et al. in 2007 [25]. This phase I trial included 44 patients, of which 11 patients had a BRCA mutation associated cancer. Dose escalation was guided by toxicity, pharmacokinetic and pharmacodynamic data. Based on the encouraging antitumor activity, many in whom had BRCA1/2 mutations, the trial was subsequently expanded to concentrate on cancers in patients with BRCA mutations and was presented in 2008 by Fong et al. [26], followed by publication of the manuscript in 2009 [27]. The drug was well tolerated in both BRCA mutated and normal populations. Most toxicities were grade 1-2 (≥95%), consisting of fatigue (28%), nausea (28%), vomiting (18%), loss of taste (13%), and anorexia (12%). Grade 3-4 toxicities were rare, consisting of myelosuppression (≤5%), nausea and vomiting (2-3%), and dizziness or mood changes (2-3%) [27]. Of the 60 patients that were enrolled and treated, 19 of 23 BRCA-positive carrriers were evaluable. 12 of the 19 (63%) had a clinical benefit from olaparib, with radiologic or tumor marker responses, or stable disease for 4 months or more [27]. Patient response was seen in those receiving a minimum of 100 mg twice daily up to 400 mg twice daily. Response was the greatest in patients with platinum-sensitive disease, although duration of response was the same regardless of the platinum-free interval [26].

Recently data was presented from a phase II study of olaparib in women with advanced EOC with known mutations in BRCA1/2 [28]. Two patient cohorts received continuous oral olaparib in 28-day cycles; 33 patients received 400 mg orally twice daily, while 24 patients received 100 mg twice daily. The choice of dosing and schedule was based on the phase I trial above [25]. The objective response rate measured by RECIST criteria was 33% at the 400 mg dose, and 12.5% at the 100 mg dose, suggesting that there may be a dose response effect. The toxicity profile was mainly mild, consisting of grade 1 or 2 nausea (44%) and fatigue (35%), with few grade 3 or 4 toxicities. Interestingly, although numbers were low, in this study there appeared to be a higher response rate in platinum resistant patients (38% versus 14%), which was opposite to that observed in the earlier phase I study (Table 1), where response was the greatest in platinum-sensitive patients. Laboratory studies have previously suggested that platinum resistant patients may reacquire BRCA function [29] thus potentially making them resistant to the effects of PARP inhibition. Taken together, the clinical data suggest that we still have a lot to learn with regard to target populations and the role of PARP inhibition. Furthermore, data from the phase II study appears to give an early indication that response (both RECIST and CA125) may be greater in those patients with BRCA2 mutations. This would be in line with the known mechanism of action of the two BRCA proteins as BRCA2 plays a key role in the repair pathway; whereas BRCA1 functions as a signaling molecule [30]. This phase II study concluded that oral olaparib is well tolerated and highly active in advanced, chemotherapy-refractory BRCA-deficient EOC, with greater activity seen at a higher dose of 400 mg twice daily. The optimal patient group with respect to platinum sensitivity has not been defined. 

Reassuringly in the clinical studies there does not appear to be an increase in toxicity between BRCA mutation carriers compared to noncarriers, supporting the theory that PARP inhibitors should not result in increased toxicity to heterozygote cells [6, 7].

These recent phase I and phase II trials are particularly promising for patients with BRCA-associated EOC. Further phase II trials are currently underway which will help further elucidate the role and potential for this new targeted therapy.

## 5. PARP Inhibitors in Sporadic Ovarian Cancers

BRCA-associated EOC is associated with only 10% of all ovarian cancers. However, loss of BRCA1/2 function is not exclusive to inheriting a mutation in the BRCA1/2 genes [31]. The results seen in known BRCA1 and 2 mutation carriers may also be relevant to the sporadic EOC patient population.

Epigenetic gene inactivation is a well-recognized phenomenon with 31% of EOC exhibiting aberrant methylation of the BRCA1 promoter [32]. Furthermore, genetic or epigenetic events occurring in other components of the HR pathway can be found in sporadic EOC [15, 33]. These tumors seem to be similar to BRCA1- or BRCA2-mutated tumors, even though they do not have mutations to either of these genes, a concept called “BRCAness.” [15, 33]. One molecular characterization study suggested that over 50% of patients with high-grade EOC had loss of BRCA function, either by genetic or epigenetic events [34]. Studies have shown that the loss of functional proteins in the HR pathway may lead these cells to be sensitive to PARP inhibition [35]. Identification of “BRCA-like” EOC populations who may benefit from this new therapy through the identification and validation of biomarkers is an active area of ongoing research.

## 6. Future Directions

At least 6 PARP inhibitors, including AG0146999 (Pfizer) and MK4827 (Merck), are under investigation either as single agents and/or in combination with other agents or treatment modalities. Phase II studies in women with advanced EOC in both BRCA1/2 mutation carriers and high-grade EOC of unknown BRCA status are ongoing, many incorporating translational research questions which are vital to our understanding of the biology of PARP inhibition. Currently, olaparib is being evaluated in a randomized phase II trial comparing this agent with pegylated liposomal doxorubicin in patients with BRCA-mutated EOC with a platinum-free interval of 0–12 months [36]. 

Early data combining PARP inhibitors with cytotoxics suggested that the combinations may be toxic and that substantial dose reductions of the cytotoxic agents may be required [37]. Intriguingly, a randomized study in women with triple negative breast cancer presented at this year's American Society of Clinical Oncology (ASCO) suggests that this may not always be the case. Patients were randomized to receive either gemcitabine 1000 mg/m^2^ and carboplatin AUC 2 on days 1 and 8 with or without the PARP inhibitor BSI-201 [38]. In this study there was no difference in the rates of toxicity or dose adjustments between the two arms. Response rates were significantly higher (*P* = .002) for women receiving the PARP inhibitor. Currently many combination studies are underway; the results are awaited with interest. Combination studies in women with both hereditary and sporadic EOC are expected in the future.

Further defining the role of PARP inhibitors in the clinic is ongoing. Olaparib is being evaluated in a randomized placebo-controlled trial as a maintenance therapy in patients with sporadic EOC at high risk of early recurrence [39]. Furthermore, some suggest that PARP inhibitors could be used to prevent cancers in patients who are BRCA mutation carriers [40]. This approach, however, requires careful consideration and some caution with the potential for the development of drug resistance in long-term use of PARP inhibitors. 

Investigation of the PARP inhibitors in the nonhereditary EOC population is very active with both the impact of treatment on patients without BRCA defects and the search for populations of women who have lost functional proteins in the HR pathway. Investigation of PARP inhibitor resistance and ways to overcome this resistance are emerging fields.

## 7. Conclusions

We are living in exciting times as our knowledge of tumor cell biology expands and new agents become available. As we move into the era of personalized medicine, the emerging data regarding the use of PARP inhibitors in patients with BRCA-associated EOC are encouraging and inspiring. Expansion and identification of further patient groups who will benefit from this approach are a priority. Over the next few years we expect to see an explosion in the publication of studies exploring the use and role of PARP inhibitors in the clinic. Careful clinical trial design, and the development and validation of biomarkers are essential if we are to make the optimal use of these exciting agents and improve outcome for women with EOC.

## Figures and Tables

**Table 1:** Responses rates of women with epithelial ovarian cancer to olaparib (AZD2281) by platinum sensitivity in Phase I (Fong et al.) [26] and Phase II trials (Audeh et al.) [28].

	No. evaluable		Responders by RECIST (%)		Responders by RECIST or GCIG (%)	
	Phase I [26]	Phase II [28]	Phase I [26]	Phase II [28]	Phase I [26]	Phase II [28]
Total	46	33	13 (28%)	11 (33%)	21 (46%)	20 (61%)
Platinum sensitive (>6 months)	10	7	5 (50%)	1 (14%)	8 (80%)	—
Platinum resistant (≤6 months)	25	26	8 (32%)	10 (38%)	11 (44%)	—
Platinum refractory	11	—	0 (0%)	—	2 (18%)	—
